# Study on anxiety and depression in elderly patients with malignant liver tumor undergoing hepatectomy

**DOI:** 10.1186/s40001-023-01040-7

**Published:** 2023-02-20

**Authors:** Lining Xu, Yingying Xu, Guiping Li, Bo Yang

**Affiliations:** 1 Department of General Surgery, The Second Medical Center & National Clinical Research Center for Geriatric Diseases, Chinese PLA General Hospital, Beijing, 100853 China; 2 Department of Internal Medicine, Henan Cancer Hospital, Zhengzhou, 450003 China; 3 Department of Radiology, Hubei Province Integrated Hospital of Chinese and Western Medicine, Wuhan, 430015 China; 4 Department of Radiology, Affiliated Union Hospital, Tongji Medical College, Huazhong University of Science and Technology, Wuhan, 430022 China

**Keywords:** Malignant liver tumor, Hepatectomy, Elderly patients, Anxiety, Depression

## Abstract

**Background:**

Patients with malignant tumors are more likely to have psychological problems due to their worries about their life expectancy. To understand the psychological status of elderly patients with malignant liver tumor undergoing hepatectomy better, the study was designed to investigate the current situation of anxiety and depression in elderly patients with malignant liver tumor undergoing hepatectomy and to analyze its related factors.

**Methods:**

A total of 126 elderly patients with malignant liver tumor undergoing hepatectomy were selected as the research objects. The anxiety and depression of all subjects were evaluated by HADS (Hosptial Anxiety and Depression Scale). The correlation factors affecting the psychological state of elderly patients with malignant liver tumor undergoing hepatectomy were analyzed by linear regression method.

**Results:**

The HADS-A score of elderly patients with malignant liver tumor undergoing hepatectomy was 8.79 ± 2.56, among which 37 patients were asymptomatic, 60 patients with suspicious symptoms, and 29 patients with definite symptoms. The HADS-D score was 8.40 ± 2.97, among which 61 patients were asymptomatic, 39 patients with suspicious symptoms, and 26 patients with definite symptoms. Multivariate analysis using linear regression method showed that FRAIL score, residence, and complication were significantly associated with anxiety and depression of elderly patients with malignant liver tumor undergoing hepatectomy.

**Conclusions:**

Anxiety and depression in elderly patients with malignant liver tumor undergoing hepatectomy were obvious. FRAIL score, regional differences, and the complication were the risk factors for anxiety and depression in elderly patients with malignant liver tumor undergoing hepatectomy. Improving frailty, reducing regional differences, and preventing complications is beneficial to alleviate the adverse mood of elderly patients with malignant liver tumor undergoing hepatectomy.

## Background

With the in-depth study of psychosomatic diseases in recent years [1], it has been found that the emotional state and psychological feelings of patients in the vast majority of clinical diseases have an important impact on the occurrence and development of diseases. With the degeneration of brain function, the elderly are more likely to have adverse psychological problems such as anxiety and depression [2, 3], which are often ignored, resulting in prolonged hospitalization, increased medical costs, and even serious impact on the life and social activities of patients and their families. The current clinical treatment mainly focuses on the operation, while ignoring the anxiety and depression of such patients, which is not conductive to the subsequent treatment and rehabilitation of patients. Patients with malignant tumors are more likely to have psychological problems due to their worries about their life expectancy [4]. To understand the psychological status of elderly patients with malignant liver tumor undergoing hepatectomy better, the study was designed to investigate the psychological status of patients with malignant liver tumor undergoing hepatectomy and analyze the related risk factors by the Hospital Anxiety and Depression Scale(HADS), so as to detect such clinical problems at an early stage and provide guidance for clinical development of relevant psychological intervention measures, improving the quality of patients’ life and shortening the length of hospital stay.

## Materials and methods

### Subjects

Elderly liver surgical patients admitted to the Chinese PLA General Hospital from January 2020 to December 2021 were selected as the research objects. Inclusion criteria:(1) Age ≥ 60 years old, (2) Malignant liver tumor undergoing hepatectomy, (3) Have good expression ability, text comprehension ability and reading ability, (4) Be informed of the research content and willing to cooperate. Exclusion criteria: (1) Combined history of mental illness, (2) Had suffered major psychological trauma before enrollment.

### Investigation methods

On the day of discharge, the enrolled patients were surveyed by questionnaire. Before the survey, all patients understood the purpose of the investigation and the consent of patients was obtained. The questionnaire were filled in by the patients according to their own actual intention. Other general information and perioperative related factors were collected by medical staff according to medical records.

### Investigation content

#### General information

Patients' gender, age, education, family income, residence, marital status, state of frailty, and number of patients in the ward were included. Frailty was assessed using the FRAIL scale.

#### Perioperative and operation related factors

A full medical history was taken from all patients and the patients were subjected to a thorough physical examination. Intra-operative data were collected from the operative notes and the anesthesia records that covered blood transfusion, estimated blood loss, and other related items.

#### Hospital anxiety and depression scale (HADS)

Hospital Anxiety and Depression Scale (HADS) compiled by Zigmond and Snaith in 1983 [5], it is mainly applied to the screening of anxiety and depression symptoms in hospitalized patients. It is composed of 14 items, including 7 items relating to depression and 7 items relating to anxiety. The scores of anxiety and depression subscales: 0–7 are considered asymptomatic, 8–10 are considered suspicious symptomatic, 11–21 are considered definitely symptomatic.

### Statistical analysis

SPSS 25.0 statistical software was used for data processing and statistical analysis. Measurement data was described as mean ± standard deviation. Univariate analysis ANOVA was used to analyze the relationship between anxiety and depression of patients with malignant liver tumor undergoing hepatectomy and perioperative factors, and multivariate analysis was performed on the related factors affecting anxiety and depression of patients with malignant liver tumor undergoing hepatectomy by linear regression. *P* < 0.05 was considered statistically significant.

## Results

A total of 126 questionnaires were sent out and effectively received. The effective recovery rate was 100%.

### Anxiety and depression of patients with malignant liver tumor undergoing hepatectomy

The HADS-A score of elderly patients with malignant liver tumor undergoing hepatectomy was 8.79 ± 2.56, among which 37 patients were asymptomatic, 60 patients with suspicious symptoms, and 29 patients with definite symptoms. The HADS-D score was 8.40 ± 2.97, among which 61 patients were asymptomatic, 39 patients with suspicious symptoms, and 26 patients with definite symptoms.

### Univariate analysis of related factors of anxiety and depression in patients with malignant liver tumor undergoing hepatectomy

Univariate analysis showed that residence, education, FRAIL score, and complication were associated with anxiety and depression in elderly patients with malignant liver tumor undergoing hepatectomy. See Tables 1, 2 and 3 for details.

**Table 1:** Univariate analysis of factors associated with anxiety and depression in elderly patients with malignant liver tumor undergoing hepatectomy

Factors	N	Percentage (%)	HADS-A		HADS-D	
			Mean ± S	*P*	Mean ± S	*P*
Gender						
Male	124	98.41	8.46 ± 2.96	0.103	8.85 ± 2.53	0.109
Female	2	1.59	5.00 ± 1.41		5.00 ± 0.00	
Marriage						
Married	121	96.03	8.69 ± 2.46	0.088	8.36 ± 2.89	0.408
Remarriage	3	2.38	11.67 ± 5.51		10.67 ± 6.43	
Widowed	2	1.59	10.50 ± 2.12		8.00 ± 1.41	
Residence						
Local	76	60.32	8.38 ± 2.29	0.025	7.96 ± 2.62	0.038
Ecdemic	50	39.68	9.42 ± 2.84		9.08 ± 3.36	
Education						
Senior high school or below	56	44.44	9.25 ± 2.53	0.000	8.84 ± 2.80	0.001
University or above	70	55.56	8.30 ± 2.29		7.93 ± 2.86	
Income						
RMB 20,000 or above per month	62	49.21	8.31 ± 2.99	0.716	8.63 ± 2.63	0.480
Less than RMB 20,000 per month	64	50.79	8.50 ± 2.99		8.95 ± 2.51	
FRAIL score						
0	41		8.17 ± 2.37	0.025	7.68 ± 2.49	0.018
1–2	53		8.68 ± 2.34		8.40 ± 2.61	
3–5	32		9.78 ± 2.92		9.34 ± 3.82	
Laparoscopic resection						
Yes	8	6.35	7.63 ± 3.46	0.184	6.50 ± 2.51	0.061
No	118	93.65	8.87 ± 2.49		8.53 ± 2.97	
Surgical history						
Yes	94	74.60	8.725 ± 2.44	0.766	8.30 ± 2.83	0.510
No	32	25.40	8.83 ± 2.63		8.69 ± 3.42	
Blood transfusion						
Yes	36	28.57	9.47 ± 2.89	0.060	8.47 ± 3.10	0.873
No	90	71.43	8.52 ± 2.38		8.38 ± 2.94	
Complication						
Yes	16	12.70	10.94 ± 3.02	0.000	10.38 ± 3.76	0.004
No	110	87.30	8.48 ± 2.35		8.12 ± 2.75	
Inpatient ward						
Single ward	56	44.44	8.68 ± 2.65	0.654	8.02 ± 2.91	0.192
Multi person ward	70	55.56	8.89 ± 2.51		8.71 ± 3.00	

^*^One way ANOVA test

**Table 2:** Univariate analysis of factors associated with anxiety in elderly patients with malignant liver tumor undergoing hepatectomy

Factors	HADS-A	Mean ± SD	*P*-Value*
Age (years)	0–7	68.00 ± 2.83	0.108
	8–10	69.10 ± 4.05	
	11–21	67.55 ± 3.09	
Volume of blood loss (mL)	0–7	396.15 ± 379.39	0.503
	8–10	681.82 ± 1013.06	
	11–21	531.82 ± 502.98	
Operating time (minutes)	0–7	188.78 ± 111.90	0.465
	8–10	199.42 ± 85.49	
	11–21	219.14 ± 108.78	
Volume of blood transfusion (mL)	0–7	138.92 ± 307.75	0.309
	8–10	252.33 ± 429.70	
	11–21	266.21 ± 412.24	
Lesion size (mm)	0–7	53.77 ± 34.90	0.469
	8–10	63.32 ± 41.89	
	11–21	53.81 ± 37.04	
Number of complication	0–7	0.03 ± 0.16	0.148
	8–10	0.20 ± 0.61	
	11–21	0.24 ± 0.51	
Total length of stay (days)	0–7	19.22 ± 9.43	0.285
	8–10	20.72 ± 8.82	
	11–21	22.97 ± 10.92	
Postoperative length of stay (days)	0–7	11.35 ± 5.75	0.221
	8–10	12.53 ± 6.05	
	11–21	14.41 ± 10.02	
Preoperative length of stay (days)	0–7	7.86 ± 6.45	0.872
	8–10	8.18 ± 4.49	
	11–21	8.55 ± 5.15	

^*^One way ANOVA test

**Table 3:** Univariate analysis of factors associated with depression in elderly patients with malignant liver tumor undergoing hepatectomy

Factors	HADS-D	Mean ± SD	*P*-Value*
Age (years)	0–7	68.56 ± 3.70	0.913
	8–10	68.26 ± 3.65	
	11–21	68.35 ± 3.17	
Volume of blood loss (mL)	0–7	574.24 ± 474.53	0.775
	8–10	687.50 ± 1302.36	
	11–21	518.18 ± 513.52	
Operating time (minutes)	0–7	208.44 ± 107.16	0.680
	8–10	190.77 ± 91.36	
	11–21	198.08 ± 92.64	
Volume of blood transfusion (mL)	0–7	215.57 ± 366.39	0.576
	8–10	186.92 ± 357.93	
	11–21	290.77 ± 503.30	
Lesion size (mm)	0–7	62.22 ± 45.21	0.597
	8–10	53.58 ± 30.95	
	11–21	60.87 ± 38.95	
Number of complication	0–7	0.13 ± 0.50	0.221
	8–10	0.10 ± 0.45	
	11–21	0.31 ± 0.55	
Total length of stay (days)	0–7	19.82 ± 8.52	0.348
	8–10	20.79 ± 9.50	
	11–21	23.08 ± 11.62	
Postoperative length of stay (days)	0–7	11.84 ± 5.48	0.094
	8–10	12.05 ± 6.20	
	11–21	15.31 ± 10.61	
Preoperative length of stay (days)	0–7	7.98 ± 5.64	0.710
	8–10	8.74 ± 5.27	
	11–21	7.77 ± 4.29	

^*^One way ANOVA test

### Multivariate analysis of anxiety and depression related factors in patients with malignant liver tumor undergoing hepatectomy

Multivariate analysis using linear regression method showed that residence, FRAIL score, and complication were significantly associated with anxiety and depression of elderly patients with malignant liver tumor undergoing hepatectomy. See Table 4 for details.

**Table 4:** Multivariate analysis of factors associated with anxiety and depression in elderly patients with malignant liver tumor undergoing hepatectomy

Factors associated with anxiety	P*	Factors associated with depression	P*
Region	0.015	Region	0.005
Complication	0.005	Complication	0.041
FRAIL score	0.026	FRAIL score	0.048

^*^Linear regression

## Discussion

The world population is aging rapidly and the average life expectancy is higher than ever before [6]. The twenty-first century is an era of an aging population, and the proportion of older persons surgical patients is increasing every year. Due to frailty, disease, mental trauma, environmental changes and other reasons, the elderly are more prone to psychological problems. In recent years, there has been an upward trend in psychological diseases of the elderly [7, 8]. With the development of medical science, more and more elderly people are undergoing surgery [9]. However, due to the large number of basic diseases and poor organ compensatory function of the elderly [10], mental problems such as delirium are more likely to occur after being hit by surgical trauma [11, 12]. Psychological intervention for surgical patients can also improve the surgical effect and prognosis [13].

Frailty is a typical feature of the elderly. Even in the early stage of frailty, the elderly is also related to the increase of emotional distress symptoms. Once the body is weak, the possibility of clinical depression and anxiety is higher. Frailty may be associated with identifying older people at risk of deteriorating mental health [14]. Frailty is also an independent predictor of high incidence of postoperative adverse events, and is significantly related to the incidence of complications, length of hospital stay, and psychological status. Good surgical effect also helps to improve the anxiety and depression of patients [15]. A study shows that for patients undergoing elective non cardiac surgery, their preoperative anxiety and depression symptoms are related to their physical weakness [16]. Another study on cardiac surgery shows that the frailty is related to the perioperative psychological state of patients undergoing cardiac surgery [17]. Therefore, frailty in elderly patients should be evaluated preoperatively, and a geriatrician should be consulted for further evaluation if necessary. However, there is no literature report on the psychological state of patients undergoing hepatectomy. This study showed that the FRAIL score was an independent risk factor for anxiety and depression in elderly patients with malignant liver tumor undergoing hepatectomy.

Inpatients, especially patients with malignant liver tumor undergoing hepatectomy, are in great need of family care [18]. Patients without visitation will suffer from obvious loneliness, which is more intense in the elderly. Loneliness in old age is extremely harmful [19]. Elderly lonely people are more likely to choose bad lifestyle and bad behaviors, which will lead to hypertension, coronary heart disease, stroke, diabetes, osteoporosis and other chronic disease or aggravate existing diseases. Elderly lonely people, often feel lonely and boring, easy to produce sad feelings or depressed mood, spirit flagging, will be transformed into serious depression. For hospitalized elderly lonely patients, the treatment effect is poor and complications are more [20]. Lack of social support exacerbates the loneliness of elderly patients [21].

For elderly patients who come from other places, most of them have no relatives to ask for help in the hospital, and they are not familiar with the hospital environment, which makes elderly patients feel more helpless. This study shows that regional differences are significant factors for anxiety and depression in elderly patients with malignant liver tumor undergoing hepatectomy. The degree of anxiety and depression of patients from other places was significantly higher than that of patients from the hospital residence.

Operation is the main stimulation for patients' abnormal psychology. The operation process, postoperative care, and anesthesia may cause neurological disorders, endocrine disorders and abnormal behavior, resulting in emotional changes of anxiety and depression. In this case, the implementation of surgery is bound to reduce the tolerance of surgery, increase the incidence of postoperative complications [22], and seriously affect the effect of surgery and postoperative rehabilitation. On the contrary, postoperative complications may also cause fear in patients, resulting in psychological problems of varying degrees, such as anxiety and depression [23]. This study showed that the complication was an independent risk factor for anxiety and depression in elderly patients with malignant liver tumor undergoing hepatectomy.

## Conclusions

In conclusion, anxiety and depression in elderly patients with malignant liver tumor undergoing hepatectomy was obvious. FRAIL score, regional differences, and the severity of complications were the risk factors for anxiety and depression in elderly patients with malignant liver tumor undergoing hepatectomy. Improving frailty, reducing regional differences, and preventing complications is beneficial to alleviate the adverse mood of elderly patients with malignant liver tumor undergoing hepatectomy.

## Data Availability

All data are from the PLA General Hospital. The data will be made available on reasonable request. The datasets generated during and/or analysed during the current study are available from the corresponding author on reasonable request.
